# Immunization with *Pneumocystis carinii* A12_1–85_ antigen activates immune function against *P. carinii*

**DOI:** 10.1186/s12865-021-00436-6

**Published:** 2021-06-27

**Authors:** Tong Tong, Zhongxin Wang, Yuanhong Xu, Jilu Shen

**Affiliations:** 1 Department of Clinical Laboratory, First Affiliated Hospital, Anhui Medical University, 218 Jixi Road, Hefei, Anhui 230022 People’s Republic of China; 2 Department of Clinical Laboratory, Fourth Affiliated Hospital, Anhui Medical University, 100 Huaihai Road, Hefei, Anhui People’s Republic of China

**Keywords:** P.carinii, A12, Immunization, Infection, PcP

## Abstract

**Background:**

*Pneumocystis* pneumonia (PcP), which is caused by *Pneumocystis carinii,* is a life-threatening infection that affects immunocompromised individuals. Unfortunately, chemoprophylaxis and dapsone are only effective for half of the patients with PcP, indicating that additional preventive methods are needed. We predicated the *pneumocystis* surface protein A12 sequence 1–85 by DNAStar software and BepiPred, and identified it as a potential vaccine candidate by bioresearch.

**Methods:**

We used recombinant A12_1–85_ as antigen to immunized mice and detected serum titer of IgG, expression of inflammatory factors by EILSA, qRT-PCR and flow cytometry.

**Results:**

Our results showed that immunization with recombinant A12_1–85_ increased the serum titer of IgG, promoted the secretion of T lymphocytes, increased the expression of inflammatory factors, and elevated lung inflammatory injury in mice.

**Conclusions:**

Our findings suggest that A12_1–85_ is a potential vaccine target for preventing *Pneumocystis carinii*. The evaluation of A12_1–85_-elicited antibodies in the prevention of PcP in humans deserves further investigation.

**Supplementary Information:**

The online version contains supplementary material available at 10.1186/s12865-021-00436-6.

## Background

*Pneumocystis* pneumonia (PcP) is a life-threatening infection caused by *Pneumocystis carinii,* with more than 400,000 cases globally [1, 2]. PcP is more likely to affect immunosuppressed people, such as cancer patients [3], organ transplant recipients [4, 5], and HIV patients [6] . Although there are drug treatments for PcP, patients with poor compliance, adverse side effects and recurrent infections remain a problem, which causes a high rate of mortality. Hence, other treatments and methods to avoid PCP warrant further investigation.

Previous studies reported that using intact *P. carinii* cells immune BALB/c mice protects against PcP [7, 8], which means that it is possible to protect against PcP using immune reactions. As *P. carinii* cannot be cultivated satisfactorily, it is important to develop a subunit vaccine. Brenda L. Tesini. et al. reported that using Pneumocystis Cross-Reactive Antigen 1 immune mice protects mice against PcP and generates an antibody against *Pneumocystis jirovecii* [9]. Jesse Wells. et al. found that BALB/c mice immunized with recombinant mouse *Pneumocystis carinii* antigen A12-thiredoxin fusion protein elevated the antibody response that recognized *P. carinii* antigen [10]. These studies indicated that vaccine-based immunotherapy could provide a novel therapeutic approach to our current management of PcP.

The A12 protein, which is homologous to *P. carinii* Kex1, was selected because it is recognized by a monoclonal antibody that provides passive prophylaxis against the development of PCP [11, 12]. This study analyzed the secondary structure, hydrophilicity, accessibility, and plasticity parameters of the A12 protein using DNAStar software(https://www.dnastar.com/t-sub-solutions-structural-biology-epitope-prediction.aspx) [13–15] and BepiPred (http://www.cbs.dtu.dk/services/BepiPred/). We found that the A12 protein has three potential B-cell antibody epitopes, which are mainly distributed in the following areas: 1–85, 92–184, 191–255. Compared with other amino acid sequences, the amino acid sequences near the N-terminal and C-terminal are usually located on the protein’s surface and have better hydrophilicity and more excellent elasticity, which are promising antigenic determinant regions. Furthermore, we entered them in BepiPred to evaluate their immunogenicity. The results showed that compared with other two amino acids sequence, the full length of A12_1–85_ was part of the linear B cell epitope. Therefore, we chose amino acids 1–85, which near the N-terminal for further study. Then, we expressed A12_1–85_ in *E. coli* and purified it. And the recombinant protein A12_1–85_ was used to immunize the mice to verify its protective efficiency.

## Result

### Recombinant A12_1–85_ immunization increased the serum titer of IgG

**Fig. 1:**
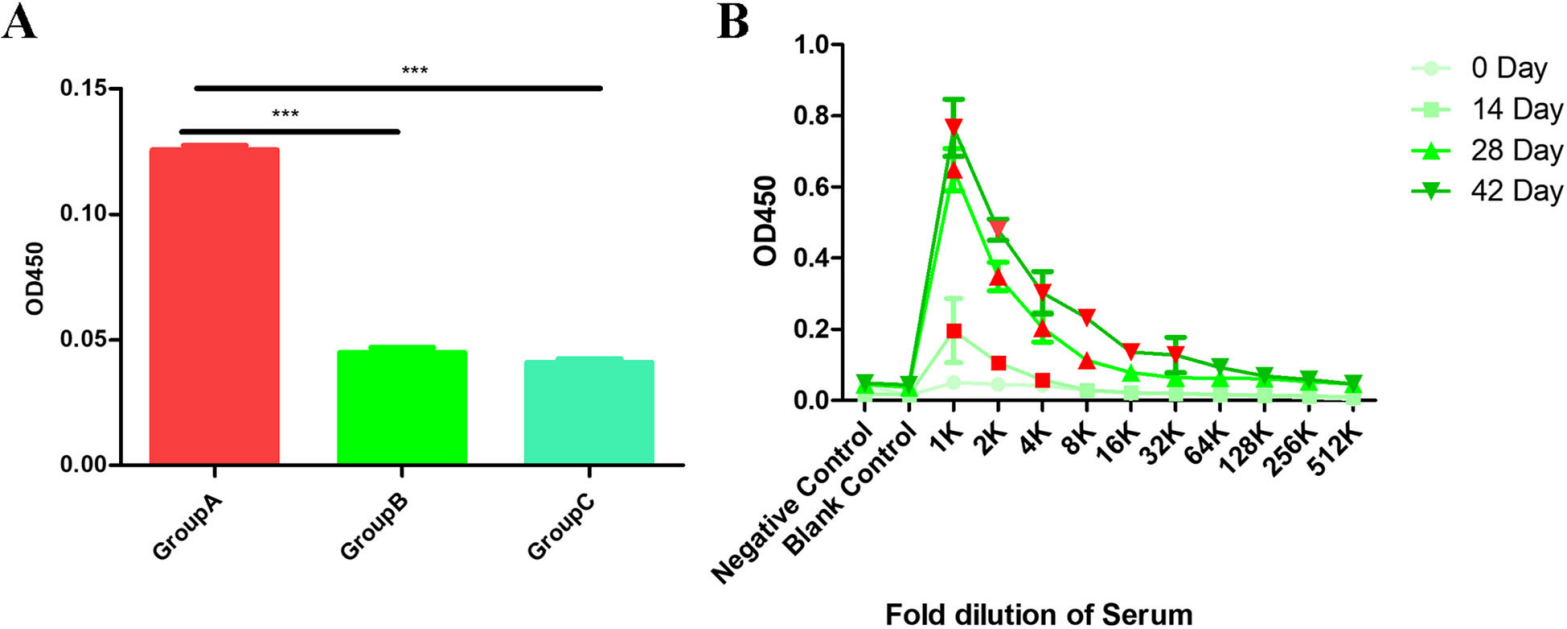
Recombinant A121–85 immunization increased the serum titer of IgG. (A) Comparison of serum titer of IgG between three groups of mice 42 days after immunization. (Group A: immunization with recombinant A121–85-adjuvant; Group B: immunization with PBS-adjuvant; Group C: immunization with PBS. *: *P* < 0.05; **: *P* < 0.01; ***: *P* < 0.001); (B) Immunized with A12_1–85_ gradually increased IgG titer with the increase of immunization times (The red plot presents effective titer)

In order to evaluate the immune effect of mice to recombinant A12_1–85_, we used ELISA to detect the serum titer of IgG between the three groups. As shown in Fig. 1, the serum IgG titer of group A was significantly higher than that of groups B and C at 42 days after immunization, which means recombinant A12_1–85_ can stimulate the body to produce a strong humoral immune response. Furthermore, we used ELISA to detect the serum titer of IgG at different times for dynamic observations of the immune effect of mice to recombinant A12_1–85_. Results showed that after secondary immunization, the serum titer of IgG reached 1:4000, after third immunization, the titer reached 1:8000, after fourth immunization, the titer reached 1:32,000, respectively, indicated that the serum titer of IgG in mice immunized with A121–85 gradually increased with the increase of immunization times(Fig. 1B).

### Immunization with recombinant A12_1–85_ promotes T lymphocytes to secrete IFN-γ

**Fig. 2:**
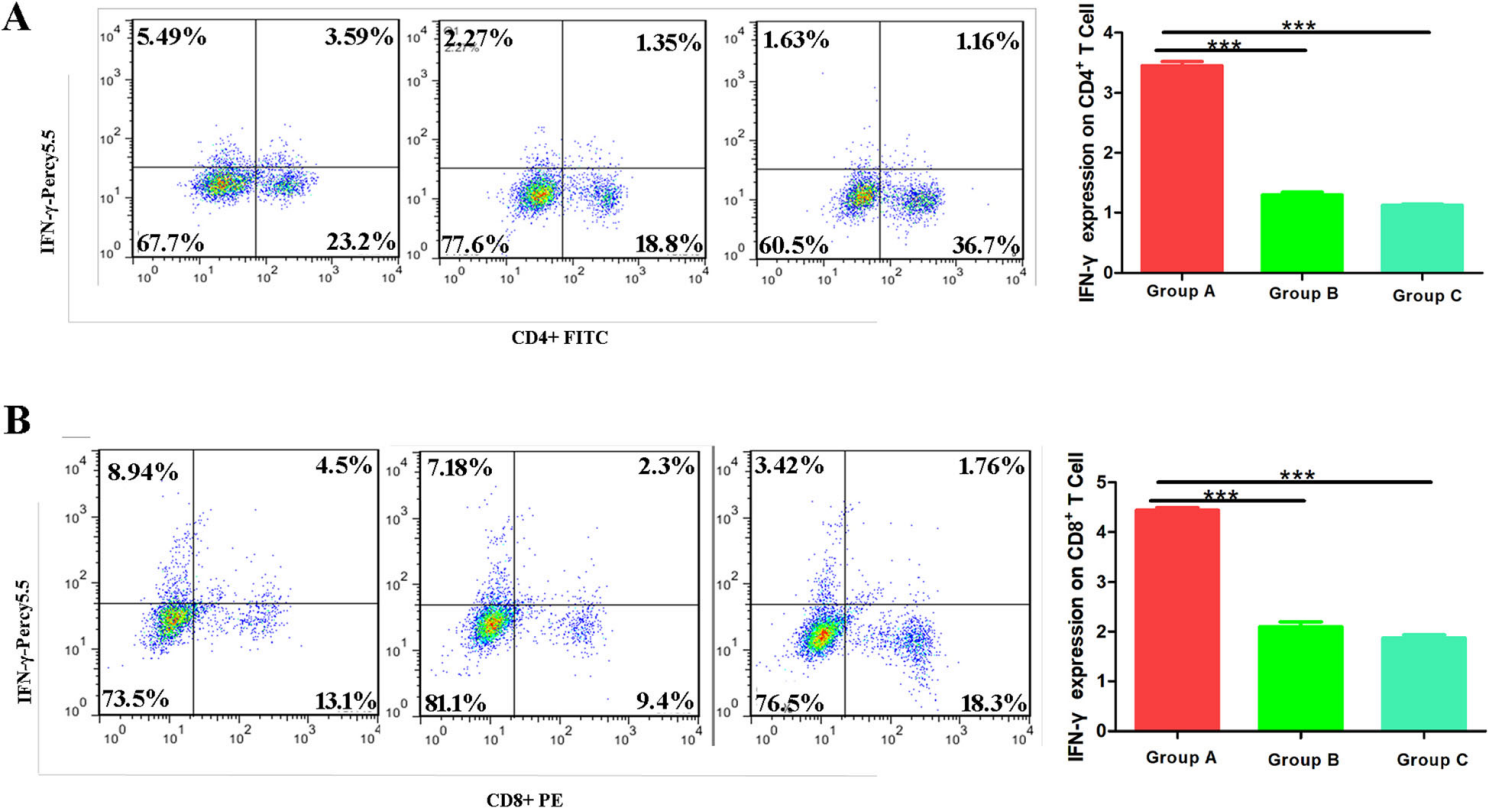
Flow cytometric analysis expression of IFN-γ on CD4+ (A)T cells and CD8 + (B) T cells of three groups. (Group A: immunization with recombinant A121–85-adjuvant; Group B: immunization with PBS-adjuvant; Group C: immunization with PBS. *: *P <* 0.05; **: *P <* 0.01; ***: *P <* 0.001)

Previous studies indicated that IFN-γsecreted by T lymphocytes plays an important role in clearing *P. carinii* [16]. Therefore, we detected IFN-γ secretions by CD4^+^ T cells and CD8^+^ T cells using FACM to evaluate the immune protective effects of recombinant A12_1–85_. As shown in Fig. 2A, the IFN-γ secreted by CD4^+^ T cells in group A(3.45 ± 0.13) was significantly higher than the group B (1.29 ± 0.08) and group C (1.12 ± 0.04)(*P* < 0.001). Meanwhile, the IFN-γ secreted by CD8^+^ T cells in group A (4.44 ± 0.1) was significantly higher than that in the group B (2.1 ± 0.18) and group C (1.9 ± 0.11)(*P <* 0.001)(Fig. 2B). These results indicated that immunization with recombinant A12_1–85_ promotes T lymphocytes secrete IFN-γ to clear P. carinii.

### Recombinant A12_1–85_ immunization promotes the expression of inflammatory cytokines

**Fig. 3:**
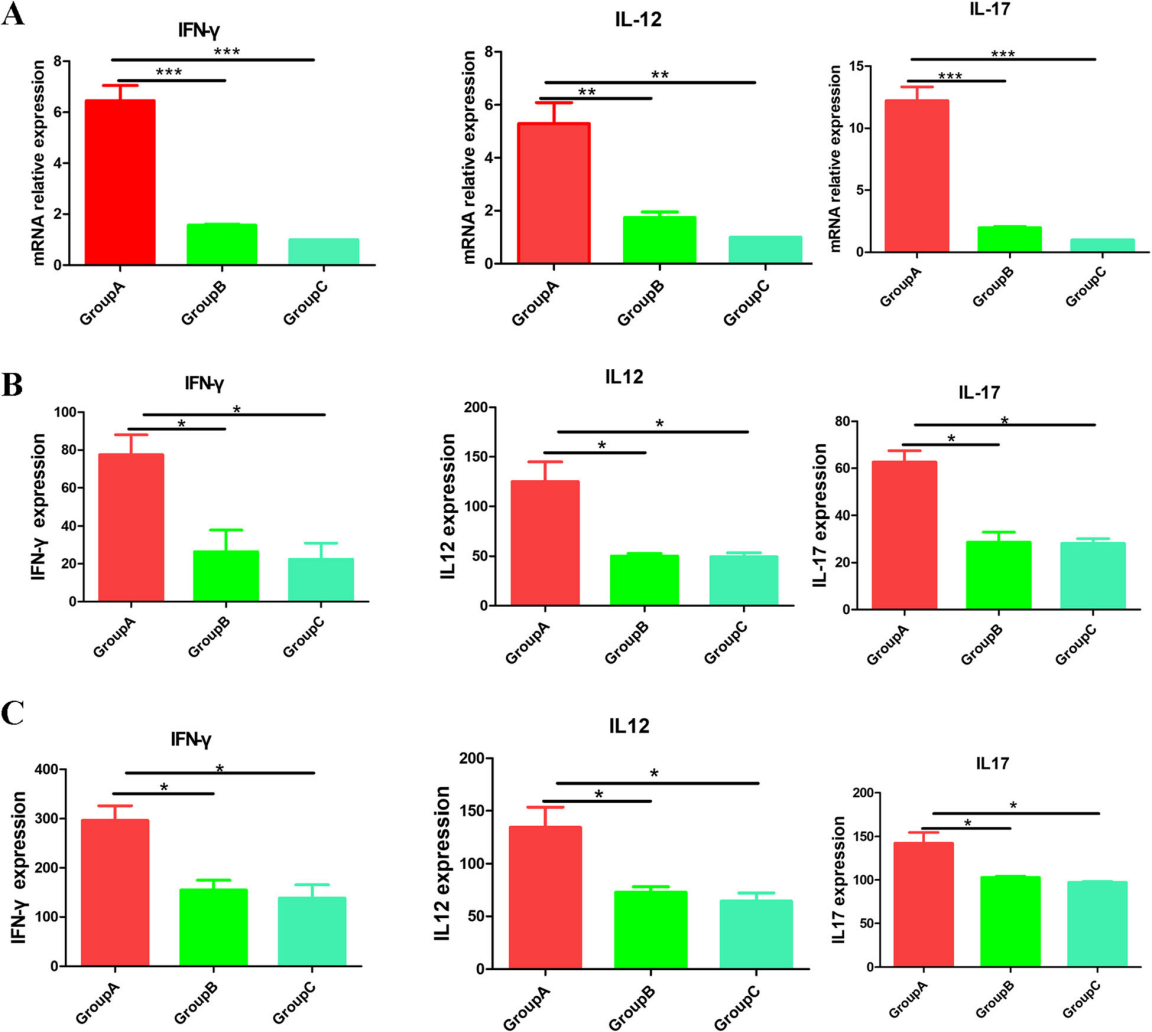
Recombinant A121–85 immunization promotes the expression of inflammatory cytokines. (A: mRNA levels of lung IFN-γ, IL-12, IL17 in three groups; B: ELISA for expression of IFN-γ, L-12, IL17 in lung tissue of mice; C: ELISA for expression of IFN-γ, L-12, IL17 in serum of mice. Group A: immunization with recombinant A121–85-adjuvant; Group B: immunization with PBS-adjuvant; Group C: immunization with PBS. *: *P <* 0.05; **: *P <* 0.01; ***: *P <* 0.001)

Studies have reported that the inflammatory cytokines IFN-γ, IL12*,* and IL17 are involved in the clearance of *Pneumocystis carinii*; therefore, we used qRT-PCR and ELISA to detect the expression of IFN-γ, IL12, and IL17 in the lung tissue and serum of mice. The qRT-PCR results showed that in the lung tissue, the mRNA levels of IFN-γ, IL12, and IL17 increased significantly in the recombinant A12_1–85_ immunization group (Fig. 3A). Furthermore, ELISA results showed that the levels of IFN-γ, IL12, and IL17 in the lung tissue and serum of recombinant A12_1–85_ immunized mice were significantly higher than that of the control groups (Fig. 3B-C). These data indicated that recombinant A12_1–85_ immunization could stimulate mice to express inflammatory factors to against *Pneumocystis carinii.*

### Immunization with recombinant A12_1–85_ significantly elevated inflammatory injury in mice

**Fig. 4:**
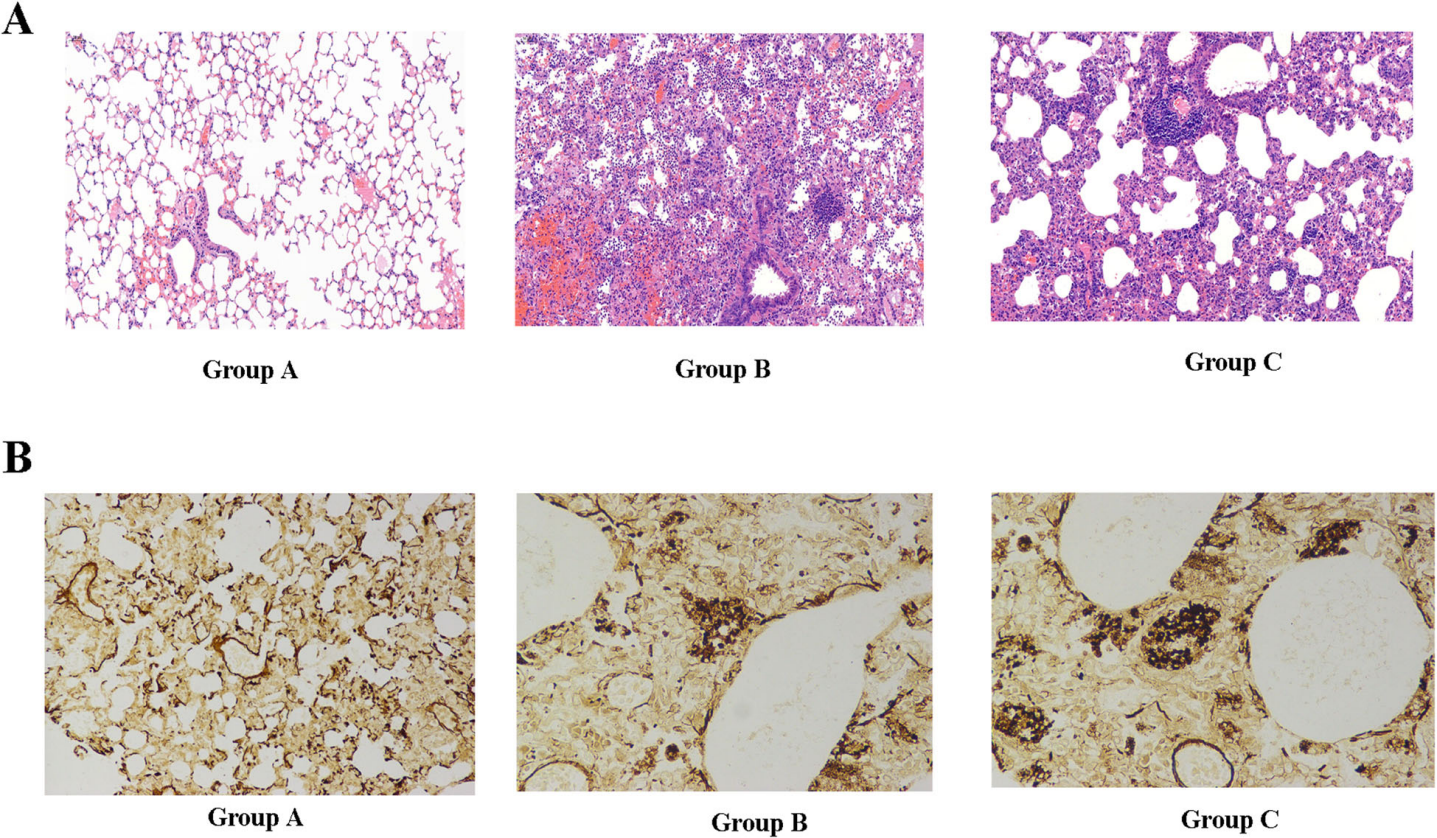
Immunization with recombinant A121–85 significantly elevated the inflammatory injury in mice. (A: HE staining for lung tissues; B: GMS staining for lung imprint. Group A: immunization with recombinant A121–85-adjuvant; Group B: immunization with PBS-adjuvant; Group C: immunization with PBS)

HE staining was performed on the lung tissue of three groups of mice to determine whether the immune response to recombinant A12_1–85_ alleviated pulmonary infections in immunosuppressed mice. As shown in Fig. 4A, the alveolar structure was clear, the alveolar walls were not significantly thickened, and the alveolar cavity was infiltrated by a moderate amount of inflammatory cells such as neutrophils and lymphocytes in the mice of group A. However, the alveolar cavities of the mice in groups B and C were filled with a large number of neutrophils and macrophages, locally accompanied by large areas of hemorrhage and some alveolar atrophy.

Furthermore, to determine whether the immune response to recombinant A12_1–85_ reduces the organism burden in immunosuppressed mice, we used GMS staining to count the number of sporangia in mouse lung imprints. The results showed that, under a microscope at 400 × magnification, the lung imprint of group A mice had a small amount of *pneumocystis*. Meanwhile, the *pneumocystis* aggregated into clusters was observed in the lung imprint of the control groups, with the number significantly higher than that of group A (Fig. 4B) (Table 1. Mean number of cysts in mice lung imprint.).

**Table 1:** Mean number of cysts in mice lung imprint

Groups	Number of mice	Mean number of cysts (per field of microscope)	*P* value(A vs B and A vs C)
A	3	0.99 ± 0.4	
B	3	5.12 ± 1.33	*P <* 0.001
C	3	5.34 ± 1.21	*P <* 0.001

## Discussion

The main targets of *Pneumocystis carinii* are patients with immune deficiencies such as AIDS, and long-term immunosuppressed patients [17, 18], that eventually developed *pneumocystis* pneumonia (PcP). There are drugs and treatments for PcP, but adverse side effects, poor compliance, and recurrent infections remain a problem. Hence, new treatments to prevent and treat PCP deserve further research.

The increasing occurrence of systemic fungal infections in humans has increased the emphasis on the production of fungal vaccines and the use of monoclonal antibodies [19]. Datta et al. reported that passive antibody transference could produce protective mAbs against specific pathogens, providing protection from infection in the absence of immunologic function [20]. De Almeida and his colleagues found that the use of an mAb against a 70-KDa glycoprotein could therapy infection with *S. schenckii* and *S. brasiliensis* in mice [21]. These studies indicated that the use of vaccines and mAb to prevent and treat fungal infections deserves further research.

As *P. carinii* cannot be satisfactorily cultivated, it is important that a subunit vaccine is developed. *P. carinii* contains a variety of surface proteins, such as surface sugars (MSG) [22], P55 [23], surface glycoprotein-related antigen (MSR) [24], and A12 [10], which increased studies have shown that these proteins are closely related to the prevention, occurrence, and development of PcP.

In the present study, we predicted the dominant epitope of A12 using a variety of molecular biology software and selected a segment of the amino acid sequence (1–85) with the dominant epitope for recombinant protein expression to study the immune characteristics.

In this study, recombinant A12_1–85_ was used to immunize BALB/c mice for the experimental group, while the immune adjuvant and PBS were used for the control groups. We found that the serum of the mice in the experimental group had higher immune titers and high titer immunoglobulins, meaning recombinant A12_1–85_ can stimulate the body to produce a strong humoral immune response to eliminate *P. carinii*. Roth JB. et al. found that the antibody against *P. carinii* disappeared in 5 HIV-infected patients with PCP during the acute infection period, while 3 patients had higher levels of anti-PC antibodies during the chronic infection period [25]. Furthermore, Kobayashi and his colleagues used rhesus monkeys infected with HIV as an animal model and found that monkeys with high IgG titers had milder symptoms of PcP [26]. These studies indicated that the humoral immune response could inhibit the infection process of *Pneumocystis*.

Studies have shown that cellular immune response and cytokines play an important role against *P. carinii* infection [27, 28]. IFN-γ, IF12, and other cytokines secreted by Th1 cells are involved in eliminating *P. carinii*. Researchers have found that the number of CD8^+^ T cells increase during *Pneumocystis* infection, which leads to a high expression of IFN-γ and is not affected by the IFN-γ antibody [28, 29]. Meanwhile, chemokines and cytokines secreted by T cells can enhance the antigen presentation of dendritic cells, accelerating the elimination of *Pneumocystis*. Thus, IFN-γ plays an important role against *P. carinii* infections. Studies have reported that infection by bacteria and fungi can stimulate macrophages, dendritic cells, and neutrophils to release IL12 [30, 31], which acts as a bridge that connects adaptive immunity and innate immunity. IL12 recruits inflammatory cells to the lungs and releases inflammatory factors against *P. carinii* [32]. IL17, which is secreted by Th17 cells, plays an important anti-inflammatory effect on the body’s lung infections, asthma, and other inflammatory reactions. Studies have shown that IL17 plays an important role in fighting fungal infections [33]. In this study, we found that the expression of IFN-γ in both the CD4 + T cells and CD8 + T cells in the experimental group was higher than that in the other two control groups. Similarly, the levels of IL12 and IL17 in the experimental group in both serum and lung tissue were also higher than those in the control groups, which indicated that immunization with recombinant A12_1–85_ could protect mice from *P. carinill*.

Finally, we used HE and GMS staining to evaluate the organism burden in the mice of the three groups. We found that the mice in the experimental group had moderate lung inflammation with few inflammatory cells infiltrating, while the mice in the control groups had relatively severe lung inflammation with widened interstitial lungs, thickened alveolar walls, and a large number of alveolar cavities. After GMS staining, the results showed that the number of cysts in the experimental group was the lowest of the groups, while the other two groups were infected with a large number of spore bacteria. The above data prove that recombinant A12_1–85_ has a powerful immune effect, which can be used as a preventive medicine or vaccine for patients with weakened immune function to reduce the incidence of PCP.

Although Jesse Wells. et al. [10] reported that mice immunized with A12_1–145_ can increase their resistance to *P. carinii* and reduce the organism burden, the A12_1–85_ we used also has immune protection against *P. carinii* and has a shorter sequence, making it more stable for vaccine development.

## Conclusion

In the present study, we found that immunization with recombinant *P. carinii* A12_1–85_ significantly increased the expression of IFN-γ, IL12 and IL17, as well as the serum titer of IgG, indicating that A12_1–85_ has a protective effect on the immunity on *P. carinii.* This study can lay a theoretical foundation for future development of *Pneumocystis* vaccines. However, the protein antigen vaccine has polymorphism and variability. In future research, we should deepen the fusion expression of A12 and other university vectors, optimize the adjuvant type, to make a composite protein vaccine to induce a substantial immune protective effect of PCP vaccine.

## Materials and methods

### Mice

Six-week-old female BALB/c mice were obtained from the Laboratory Animal Center of Anhui Medical University, China. Animal experimental procedures were approved by the Animal Ethics Committee of Anhui Medical University (No. 20200938). All of the animals received subcutaneous injections of dexamethasone (0.5 mg/mice) in the groin every 3 days for 8 weeks for established Pneumocystis infection model [16]. A conventional colony provided the mice ample environmental exposure to P. carinii and other rodent viruses. Tetracycline hydrochloride (1 mg/mL) was added to the water to control secondary bacterial infections.

### Immunization with recombinant A12_1–85_ protein

Mice were divided into a recombinant A12_1–85_- adjuvant experimental group (group A), PBS-adjuvant group (group B), and PBS group (group C), with 15 mice per group. They received multiple subcutaneous immuniOn the 42nd day, the mice were sacrificed by intraperitoneal injection of pentobarbital sodium(200mg/kg), and the spleen and lung were taken for follow-up experiments.zations every 2 weeks for a total of 4 times in the back and groin. Blood was collected by tail cutting on days 0, 14, 28, and 42 for the titer test by ELISA. On the 42nd day, the mice were sacrificed by intraperitoneal injection of pentobarbital sodium (200mg/kg), and the spleen and lung were taken for follow-up experiments.

### ELISA for serum IgG titer

The protein stock solution (600 μg/ml) was purified by recombinant A12_1–85_ and diluted to 10 μg/ml with a coating buffer. It was added to a blank ELISA plate at 200 μl per well, left overnight at 4 °C and the coating solution was discarded. Then, each well of the coated ELISA plate was added to PBS-T with 200 μl, washed, dried with filter paper, and repeated 5 times. It was blocked with 1% BSA, 100 μl per well, at 37 °C for 1 h. Each serum sample from the tail blood is 1:1000, 1:2000, 1:4000, 1:8000, 1:16000, 1:32000, 1:64000; 1:128000; 1: 256000, 1:512000 dilution, respectively, with 100 μl/well for incubating at 37 °C. The OD value was measured at 450 nm absorbance and the cutoff value is OD of negative control * 2.5.

### Flow cytometry for the detection of inflammatory factors

The immunized mice spleen was taken aseptically to prepare a single cell suspension, incubated in a 24-well plate (1 × 10^6^/well), and stimulated with the Pharmaceutical Manufacturers Association (PMA) + Ionomycin (Ion) + Brefeldin A (BFA) for 5 h in the cell incubator. Next, the cells were collected by centrifugation, the antibodies were labeled, and they were tested using FCM.

### Quantitative real-time PCR (qRT-PCR) for mRNA of inflammatory factors

The TRIzol reagent (Ambion, Austin, TX, USA) was used to extract the total RNA from the lung tissue samples according to the manufacturer’s protocol. An Advantage RT-PCR Kit and random primers were used to synthesize cDNA (Clontech, Takara, Japan). A qRT-PCR was conducted on the LightCycler 480 Detection System with SYBR Green dye (Clontech, Takara, Japan). The specific primers for the inflammatory factors were as follows: IFN-γForward: 5′- AGCAAGGCGAAAAAGGATGC-3′; IFN-γReverse: 5′-TCATTGAATGCTTGGCGCTG-3′; IL12 Forward: 5′-GATGTCACCTGCCCAACTG-3′; IL12 Reverse: 5′-TGGTTTGATGATGTCCCTGA-3′; IL17 Forward: 5′-CTCCAGAAGGCCCTCAGACTAC-3′; IL17 Reverse: 5′-GGGTCTTCATTGCGGTGG-3′; GAPDH Forward: 5′-CAACTTTGGCATTGTGGAAGG-3′; GAPDH Reverse: 5′-ACACATTGGGGGTAGGAACAC-3′.

The reaction parameters included a denaturation program (30 s at 95 °C, 1 cycle), followed by an amplification and quantification program over 40 cycles (5 s at 95 °C and 20 s at 60 °C). Each sample was tested in triplicate, and each underwent a melting curve analysis to check the specificity of amplification.

### Hematoxylin-eosin (HE) staining and Gomori’s methenamine silver(GMS) staining

A piece of lung tissue less than 0.5 cm was taken from each mouse and put into 10% formalin fixative. Water was removed with alcohol, and then xylene was added. It was then embedded in paraffin and sliced. HE or GMS were used to stain according to the manufacturer’s protocol, then observed under a microscope.

### Statistical analysis

Statistical analyses were performed using t-tests and one-way analysis of variance (ANOVA-Bonferroni test). All data were in accordance with normal distribution (supplement materials -normal distribution test data: Supplementary Fig 1-Fig 3). All statistical analyses were conducted using SPSS 16.0 (SPSS Inc., Chicago, IL, USA). A value of *p* < 0.05 was considered statistically significant.

## Supplementary Information

**Additional file 1: Supplementary Fig. 1**. The normal distribution test of the Fig. 1A data. **Supplementary Fig. 2**. The normal distribution test of the Fig. 2 data.(A. Figure 2A data; B. Figure 2B data). **Supplementary Fig. 3**. The normal distribution test of the Fig. 3 data.(A. Figure 3A data; B. Figure 3B data; C. Figure 3C data).

## Data Availability

The datasets used and/or analysed during the current study are available from the corresponding author on reasonable request.
