# Supplementary material for: Immunization with Pneumocystis carinii A121–85 antigen activates immune function against P. carinii
Source: BMC Immunol. 2021 Jun 27;22:40. doi: 10.1186/s12865-021-00436-6 (PMC8236001; doi:10.1186/s12865-021-00436-6)
Supplement: Supplementary file 1 — Additional file 1: Supplementary Fig. 1. The normal distribution test of the Fig. 1A data. Supplementary Fig. 2. The normal distribution test of the Fig. 2 data.(A. Figure 2A data; B. Figure 2B data). Supplementary Fig. 3. The normal distribution test of the Fig. 3 data.(A. Figure 3A data; B. Figure 3B data; C. Figure 3C data). [file 12865_2021_436_MOESM1_ESM.docx]

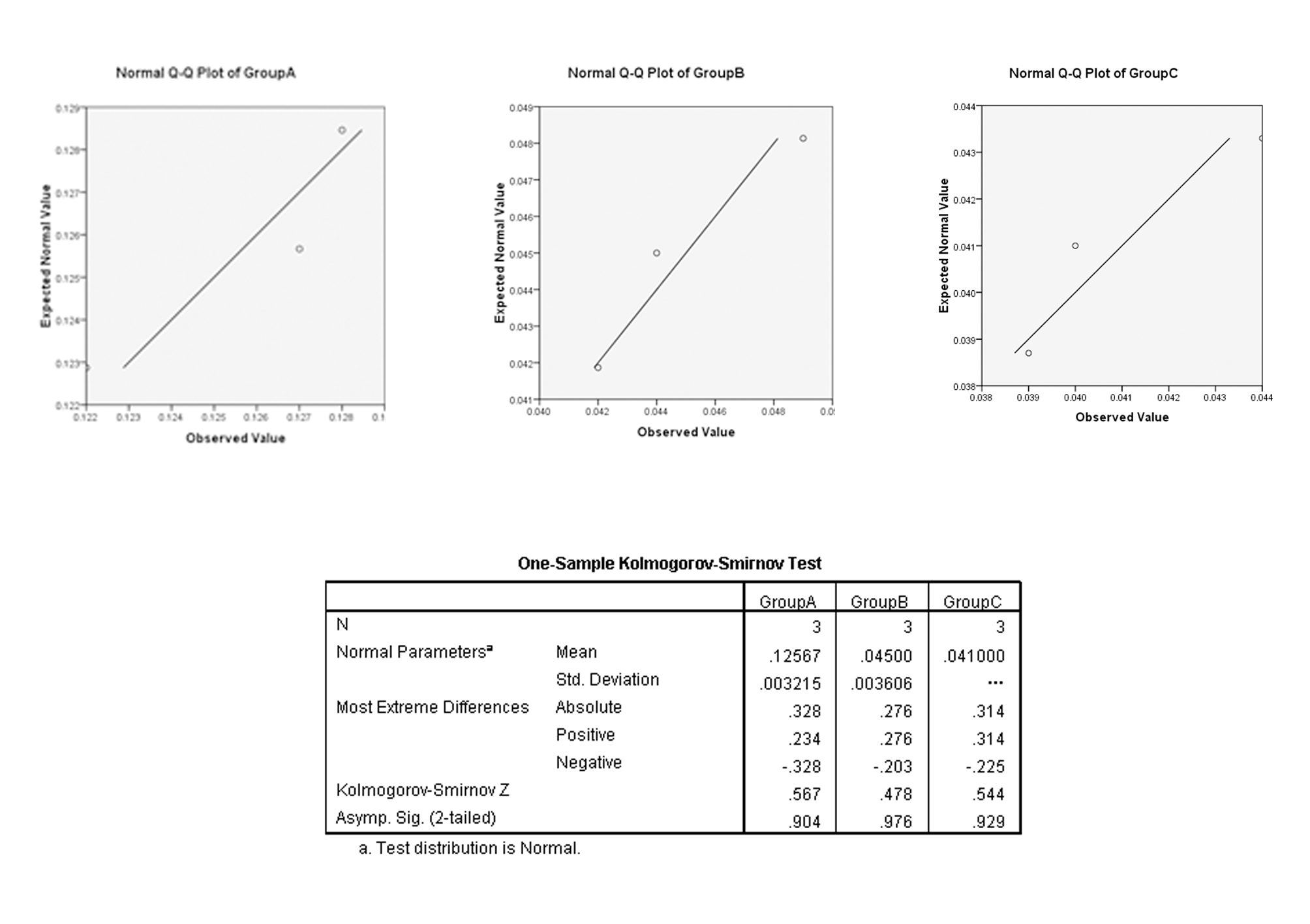


Supplementary Fig 1. The normal distribution test of the figure 1A data.


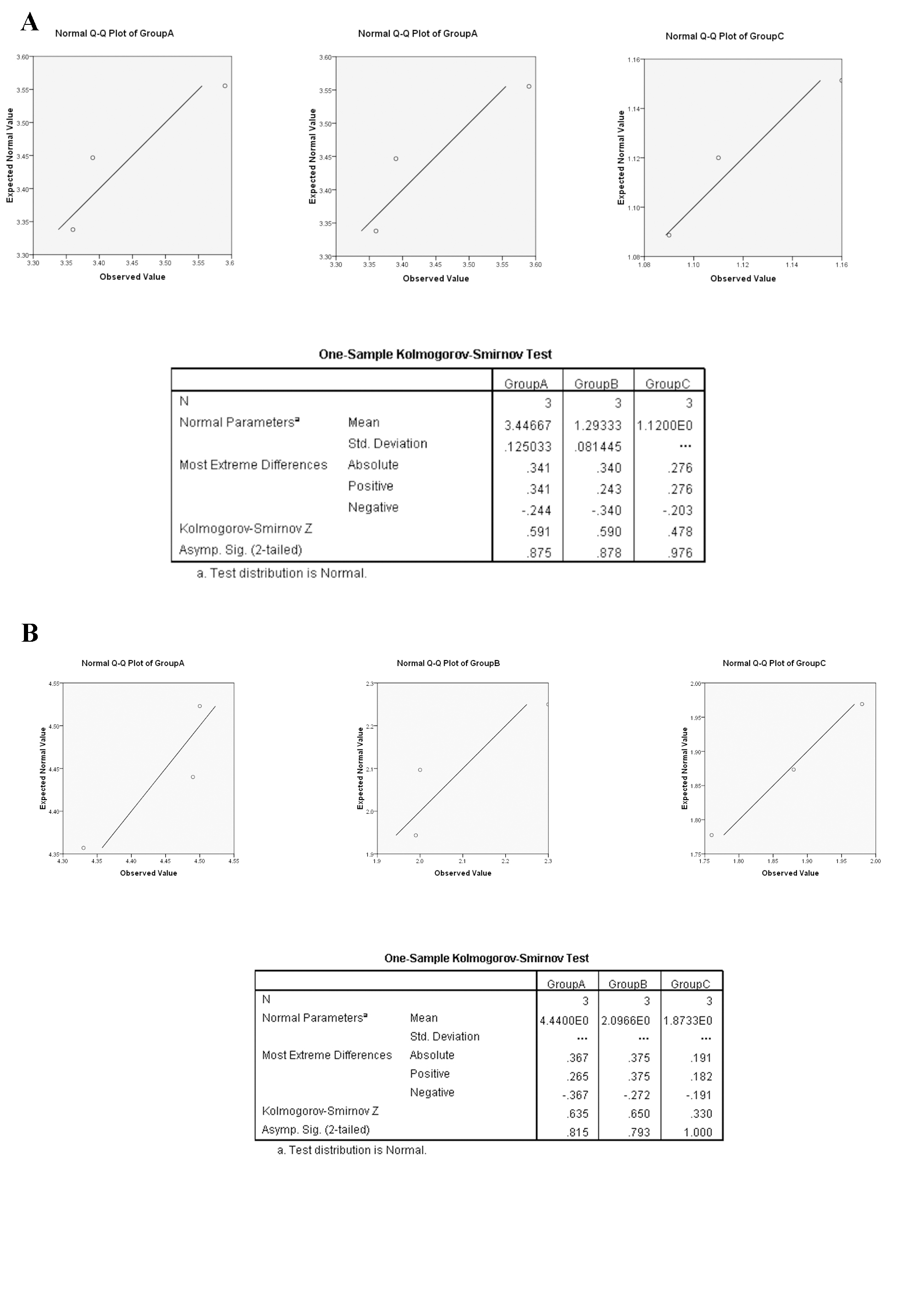


Supplementary Fig 2. The normal distribution test of the figure 2 data.(A. Figure 2A data; B.Figure 2B data)


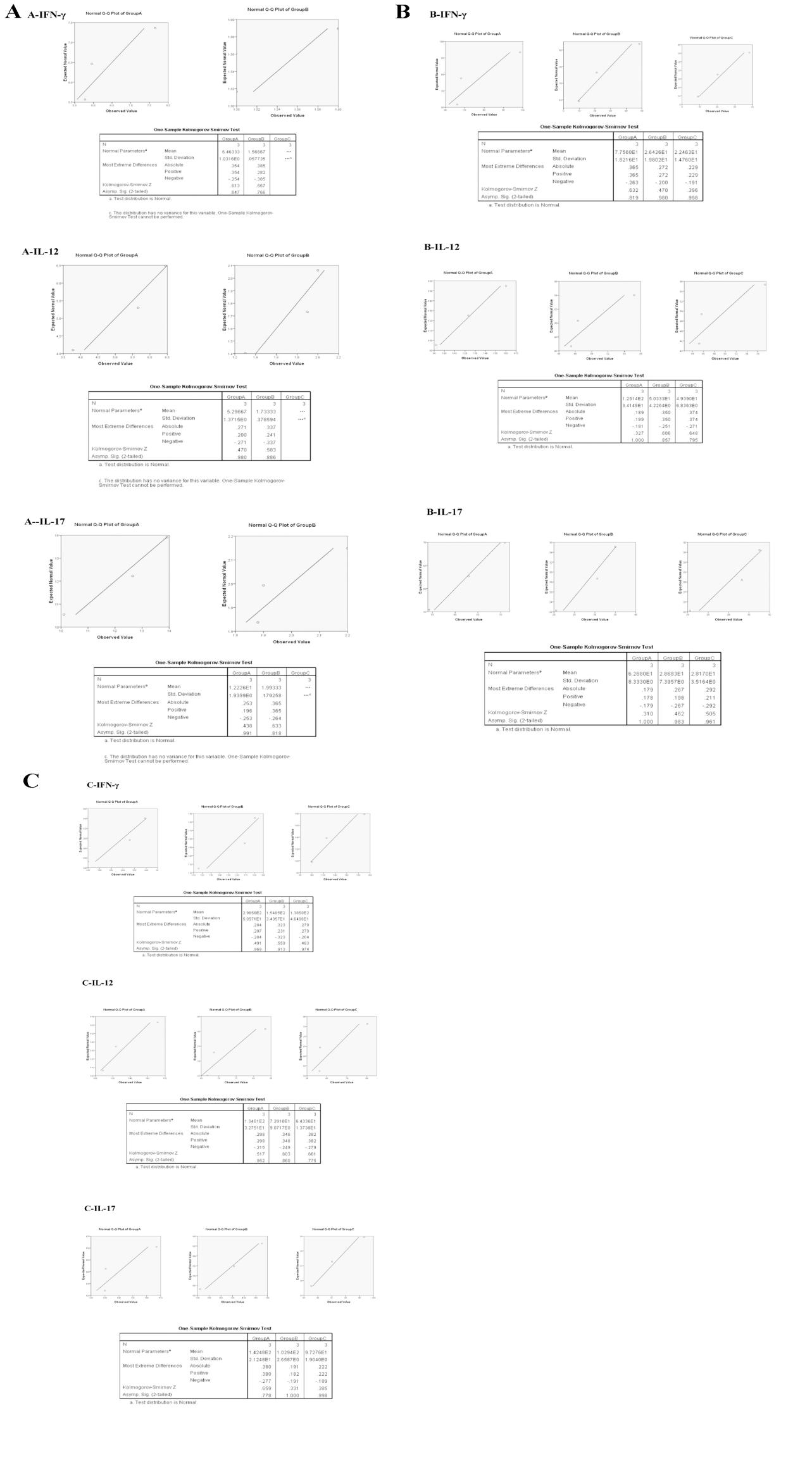


Supplementary Fig 3. The normal distribution test of the figure 3 data.(A. Figure 3A data; B.Figure 3B data; C.Figure 3C data)
